# Membrane Distillation–Crystallization for Sustainable Carbon Utilization and Storage

**DOI:** 10.1021/acs.est.3c04450

**Published:** 2023-10-19

**Authors:** Kofi S. S. Christie, Allyson McGaughey, Samantha A. McBride, Xiaohui Xu, Rodney D. Priestley, Zhiyong Jason Ren

**Affiliations:** † Andlinger Center for Energy and the Environment, Princeton University, Princeton, New Jersey 08544, United States; ‡ Department of Civil and Environmental Engineering, Princeton University, Princeton, New Jersey 08544, United States; § Department of Chemical and Biological Engineering, Princeton University, Princeton, New Jersey 08544, United States; ∥ Department of Mechanical and Aerospace Engineering, Princeton University, Princeton, New Jersey 08544, United States; ⊥ Princeton Institute for the Science and Technology of Materials, Princeton University, Princeton, New Jersey 08544, United States

**Keywords:** membrane crystallization, carbon dioxide, carbonate minerals, flue gas, amine solution, scrubbing, wastewater

## Abstract

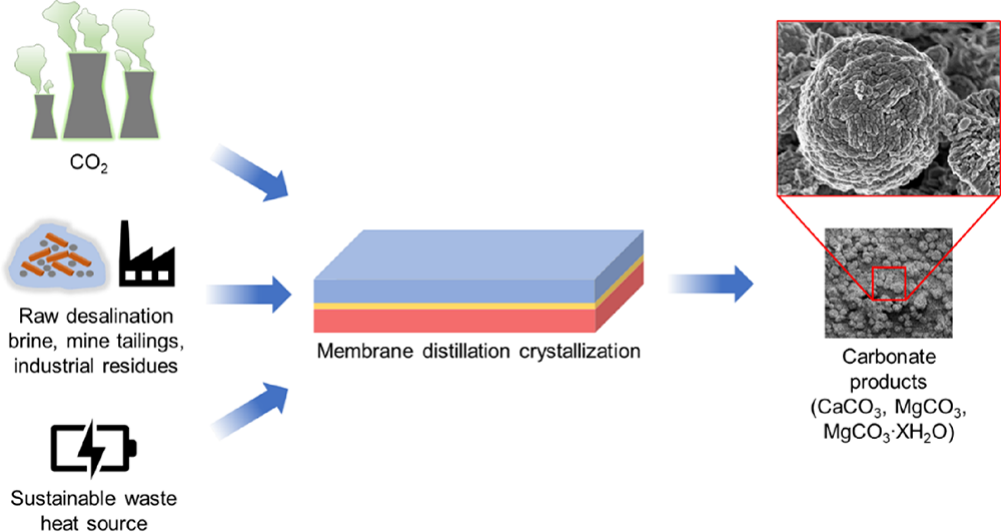

Anthropogenic greenhouse gas emissions from power plants can be limited using postcombustion carbon dioxide capture by amine-based solvents. However, sustainable strategies for the simultaneous utilization and storage of carbon dioxide are limited. In this study, membrane distillation–crystallization is used to facilitate the controllable production of carbonate minerals directly from carbon dioxide-loaded amine solutions and waste materials such as fly ash residues and waste brines from desalination. To identify the most suitable conditions for carbon mineralization, we vary the membrane type, operating conditions, and system configuration. Feed solutions with 30 wt % monoethanolamine are loaded with 5–15% CO_2_ and heated to 40–50 °C before being dosed with 0.18 M Ca^2+^ and Mg^2+^. Membranes with lower surface energy and greater roughness are found to more rapidly promote mineralization due to up to 20% greater vapor flux. Lower operating temperature improves membrane wetting tolerance by 96.2% but simultaneously reduces crystal growth rate by 48.3%. Sweeping gas membrane distillation demonstrates a 71.6% reduction in the mineralization rate and a marginal improvement (37.5%) on membrane wetting tolerance. Mineral identity and growth characteristics are presented, and the analysis is extended to explore the potential improvements for carbon mineralization as well as the feasibility of future implementation.

## Abstract 2

Membrane-facilitated carbon mineralization benefits from optimizing membrane characteristics, separation configuration, feed solution temperature, and feed solution composition.

## 1 Introduction

Global greenhouse gas emissions are linked to negative long-term climate outcomes, and capturing potentially harmful gaseous products before they enter the atmosphere is of the utmost concern.^1^ It has become increasingly important to investigate new methods for reducing and managing the emissions of greenhouse gases, including methane (CH_4_), nitrous oxide (N_2_O), and fluorinated gases, but especially carbon dioxide (CO_2_). A large portion of these methods can be classified as carbon capture, utilization, and storage (CCUS) processes, and carbon mineralization is of particular interest for simultaneous storage and utilization of CO_2_.^2−4^ For example, in a roadmap designed by the South Korean government to identify key technologies to realize a carbon-neutral society by 2050, carbon mineralization-related strategies are one of the five major technological categories identified.^5^ Altogether, the carbon mineralization of ultramafic rocks, mine tailings, alkaline industrial residues, construction and demolition waste, municipal solid waste, and naturally occurring minerals can offset up to 30 Gt of CO_2_ per year.^6^

The traditional approach to carbon mineralization has been to transfer captured CO_2_ into underground geological formations (i.e., saline aquifers, depleted oil and gas fields, and unmineable coal seams) to form carbonate minerals from suitably reactive and naturally occurring mafic and ultramafic rocks, such as wollastonite (CaSiO_3_), olivine (Mg_2_SiO_4_), and serpentine (Mg_3_(OH)_4_Si_2_O_5_).^7−12^ This approach is relatively cheap when it is considered separately from CO_2_ capture, separation, and transportation. Despite complications associated with long-term monitoring, verification of mineralization, and permanence of CO_2_ sequestration,^13^ several successful pilot operations have been carried out in Iceland,^14^ Oman,^15^ and the United States.^16^

Due to the limited availability of suitable geologic reservoirs for *in situ* geological mineralization, *ex situ* approaches have been investigated in which the weathering of alkaline earth metal silicates, oxides, and hydroxides by aqueous CO_2_ is simulated under controlled temperature, pressure, and reactor composition.^17−19^ The alkaline earth metal-bearing mineral precursors required for *ex situ* mineralization are typically derived from waste products from industrial processes, such as coal combustion fly ash, metal processing slag, cement kiln dust, and mine tailings.^20^ The advantages of the *ex situ* carbon mineralization approach include (i) valorization of solid waste products via production of carbonate salts that can be used as construction materials, food and drug additives, and paint and chemical stabilizers, (ii) high surface area waste powders maximizing the energy released from the exothermic carbonation reaction, thereby potentially offsetting energy consumption costs through utilization of the released heat, and (iii) high sequestration capacity and relatively low transportation costs due to the wide availability of suitable solid wastes near point sources of CO_2_ emissions.^6,16^ Carbon mineralization is among the most competitive CO_2_ management strategies (Table S1).^21,22^

Previous *ex situ* carbon mineralization approaches have involved dissolving CO_2_ in water, dissolving calcium- or magnesium-bearing solids to release divalent cations, and precipitating solid carbonates. The CO_2_ mineralization steps (i.e., CO_2_ dissolution, cation separation, and carbonate mineralization) can be either separated into distinct unit operations or combined for process intensification. When separated into multiple steps, optimizations of the time scales of the individual absorption and crystallization reactions are required to maximize production capacity. Additional monitoring equipment and machinery are therefore needed to synchronize each stage for the process to operate in a continuous mode. CO_2_ dissolution in water for multiple-step mineralization often needs to be catalyzed, and previous reports have used bioinspired catalysts such as carbonic anhydrase to accelerate the process.^23,24^ Similarly, to accelerate the dissolution of calcium- or magnesium-bearing solids, chelating agents like ethylenediaminetetraacetic acid (EDTA), weak acids like citric acid or acetic acid, strong acids like nitric acid or sulfuric acid, and reagents such as ammonium bisulfate have been used.^20,25−27^ In contrast, single-step approaches, such as aqueous amine looping, can eliminate these confounding factors. In aqueous amine looping, the CO_2_-loaded solvent is directly mixed with calcium- or magnesium-bearing solids for concurrent precipitation of solid carbonates and regeneration of the CO_2_-capturing solvent.^19,28−31^ Single-step processing eliminates the necessity of acids and reagents that are required for multistep processing, thereby significantly reducing scale-up costs.^32^ However, traditional single-step carbon mineralization precludes the ability to adjust the solution properties (i.e., solute concentration, solvent ratio, and residence time in proximity to the nucleating surface) during mineralization. It is well known that solution properties have a great influence on crystal morphology, size, and orientation.^33−36^ Therefore, tuning the crystallization process can enable the control and maximize the efficiency of carbon mineralization.

Crystallization-based separations operate by establishing supersaturation of the target solute to initiate nucleation and growth of solid crystals from the bulk solution.^37−42^ The saturation level of the target solute can be controlled by regulating the conditions of the solution (i.e., solution temperature, solvent removal rate, and chemical reactions between the solutes) throughout the crystallization process.^41,43^ The size, growth rate, morphology, and the crystal formation pathway (i.e., whether crystal nucleation occurs heterogeneously or homogeneously) of the targeted crystals are important in determining the final crystal properties (dissolution behavior, stability during storage under humid conditions, particulate flow properties, aesthetics, etc.), and precise control of the crystallization system is crucial for the robust and reproducible attainment of crystalline products in a sustainable way.^44^

Membrane distillation–crystallization (MDC) is an emerging method that can outperform conventional crystallization processes in both the controllability of crystallization and in the quality of the crystalline product.^45−48^ In MDC, a vapor pressure gradient is imposed across a microporous and hydrophobic membrane to induce simultaneous solvent volatilization and solute concentration before the solution enters the crystallizer.^49−52^ MDC leverages the vapor pressure gradient to precisely control the solute concentrations both spatially and temporally, thereby enabling the generation of specific crystalline products.^53−55^ MDC benefits from the low operating pressure and modular design of stand-alone membrane distillation (MD), which has gained recognition as a sustainable separation process when low-grade waste heat or renewable energy resources are utilized to drive the vapor pressure gradient. Sweeping gas membrane distillation (SGMD) is a less common MD configuration, in which flowing air is swept across the distillate side of the membrane. SGMD is advantageous for heat utilization efficiency because convective heat losses into the distillate stream are minimized.^56,57^ This is an important consideration because the duration of active mass transfer, or the feed solution concentration rate, in the MDC process is limited by the amount of heat available in the system.

**Figure 1:**
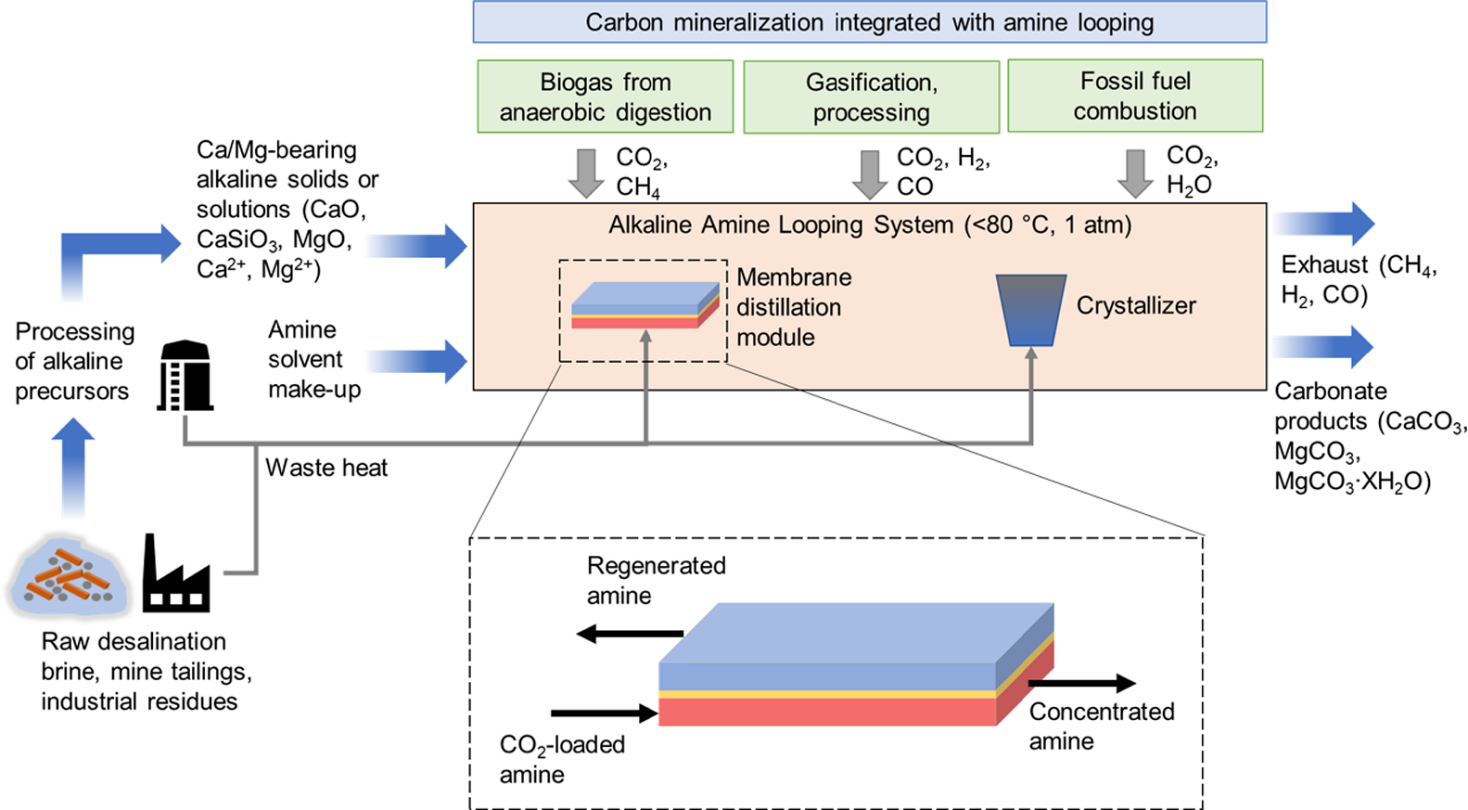
Schematic illustration of the proposed membrane-assisted carbon mineralization system with alkaline amine looping. First, alkaline earth metal ions are supplied from industrial residues and processed for mineralization. Then, the alkaline earth metal ions are combined with CO_2_-loaded amine scrubbing solution at low temperature (<80 °C) and ambient pressure (1 atm). A membrane distillation (MD) module is then employed to facilitate the precise control of the saturation state of the carbon mineralization solution. Once the combined solution is at the appropriate saturation state, the solution is directed toward a crystallizer, where carbonate minerals (CaCO_3_, MgCO_3_, and MgCO_3_*XH_2_O) are precipitated. Finally, the regenerated (lean) amine solution is recycled for further CO_2_ capture from the nearby point sources (e.g., fossil fuel combustion, gasification, and anaerobic digestion), and the process is repeated until alkaline precursors are depleted. The membrane distillation–crystallization (MDC) process involves a microporous and hydrophobic membrane that separates the heated feed stream (CO_2_-loaded amine) and an opposing stream with lower vapor pressure. Due to the vapor pressure gradient, the solvent evaporates at the feed-membrane interface and the vapor travels through the membrane pores, thereby concentrating the solutes within the feed stream and enabling precise control of the saturation state of the feed constituents and the subsequent nucleation and growth of crystals.

Previous studies have explored the influence of membrane properties, operational controls, and feed solution composition on the performance of MDC operations.^58−70^ Similarly, many studies have explored the influence of antiscalants, membrane surface coatings, and operational controls on scaling, which is an undesirable side effect of the brine concentration that can damage system performance, in stand-alone MD.^71−79^ Surprisingly, the insights gained at the intersection of membrane-based separations and crystallization^80−83^ have not previously been applied to carbon mineralization or CCUS. There is a need to expand the capability of *ex situ* carbon mineralization to better control the crystallization process and to maximize the capability of carbon mineralization. Integrating MDC into an aqueous amine looping scheme (Figure 1) is a promising prospective route to carry this out.

In this study, we use a bench-scale representation of an aqueous amine looping scheme to optimize the MDC for carbon mineralization. Several operational parameters, including membrane properties, feed solution temperature, MDC configuration, CO_2_ load, metal ion concentration, and metal species, are investigated. We compare the effect of each operational parameter on carbonate crystal growth rate and membrane wetting by a synthetic amine-based CO_2_ capture fluid stream and discuss the underlying phenomena that are potentially responsible for the varied outcomes. We also evaluate the morphological and chemical characteristics of the carbonate minerals obtained, present an environmentally friendly membrane modification procedure for improved performance, and discuss the potential and feasibility of MDC for expanded carbon mineralization application.

## 2 Materials and Methods

### 2.1 Chemicals and Membranes

Calcium chloride (CaCl_2_), magnesium chloride (MgCl_2_), monoethanolamine (MEA), and coconut oil-derived fatty acids were purchased from Thermo Fisher Scientific. Each salt and solvent was used as received without further purification. Industrial-grade CO_2_ gas was purchased from Airgas (Radnor, PA). Stock solutions of 1 M CaCl_2_, 1 M MgCl_2_, 1 M NaCl, and 30 wt % MEA were created and filtered (0.22 μm cellulose acetate) before being stored in a refrigerator until use.

Commercial poly(vinylidene fluoride) (PVDF) membranes with a nominal pore diameter of 0.45 μm were purchased from GE Healthcare (Chicago, IL). Commercial poly(tetrafluoroethylene) (PTFE) membranes with a nominal pore diameter of 0.45 μm were purchased from Sterlitech (Kent, WA).

### 2.2 Membrane Modification and Characterization

**Figure 2:**
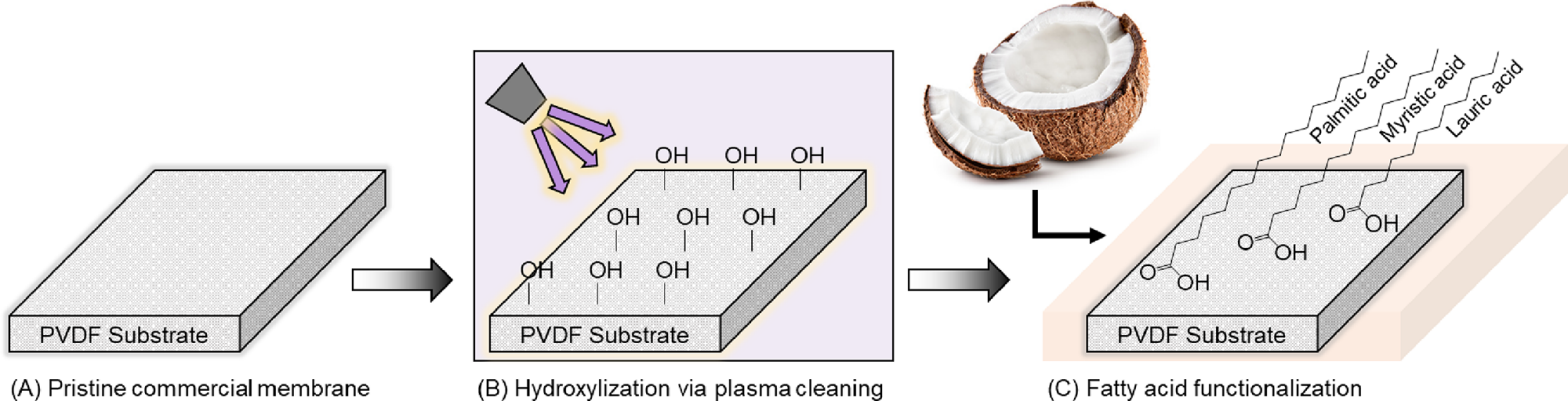
Schematic illustration of the enhanced hydrophobicity modification process. (A) The commercial PVDF membrane was first prepared for modification. (B) The surface of the PVDF membrane was then hydroxylized using plasma cleaning. (C) Coconut oil-derived fatty acids were coated on the membrane substrate via covalent bonding in a liquid phase reaction with ethanol.

Membranes with enhanced hydrophobicity were fabricated by coating them with a coconut oil-derived fatty acid. A three-step method was used to modify the commercial PVDF membrane (Figure 2). First, the membrane was subjected to plasma cleaning (Harrick Plasma PDC-001) to generate hydroxide (OH^·^) radicals on the surface.^84^ The membrane was then immersed in a 4 wt % solution of coconut oil in ethanol where the hydrophobic heads of the coconut oil fatty acids bond covalently to the radicals generated on the membrane surface.^85^ Finally, the modified membrane was subjected to heat treatment in a drying oven overnight to volatilize any remaining ethanol. The modified membrane was stored in deionized (DI) water until experimentation.

The surface morphology of each membrane was characterized by using scanning electron microscopy (SEM, Quanta 200). The surface roughness of each membrane was characterized by using atomic force microscopy (AFM, MFD-3D, Asylum Research, Santa Barbara, CA). The static contact angle of both DI water and the MEA solution was measured with an optical goniometer (Ramé-Hart Model 500). After crystallization experiments, elemental mapping of the resultant crystal species was carried out with the energy-dispersive X-ray (EDX) detector attachment of the SEM. Additionally, the identity of the crystal layer formed on the membrane surfaces was characterized by using X-ray powder diffraction (XRD, Bruker D8 Discover) and Fourier-transformed infrared spectroscopy (FTIR, Cary 630).

### 2.3 Experimental Setup for Membrane Distillation–Crystallization

A custom-made flow cell for a flat sheet membrane was used to carry out the experiments in this study (Figure S1). Peristaltic pumps were used to circulate the feed solution and the distillate through the flow cell on either side of the microporous membrane. The temperatures of the feed and distillate streams were adjusted using digitally controlled constant-temperature baths (Polystat Recirculator, Cole-Parmer, Vernon Hills, IL) and monitored using digital temperature probes (J-Type thermocouple, National Instruments, Austin, TX) at the inlet and outlet of both streams. The distillate conductivity was measured over time by using a conductivity probe (Atlas Scientific, Long Island City, NY). The vapor flux was monitored by calculating the mass of vapor transferred through the membrane using distillate mass measurements over time with a benchtop balance (Valor 7000, Ohaus, Parsippany, NJ), following eq 1:
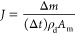
1where *J* is the water flux, Δ*m* is the mass of the distillate water collected over time (Δ*t*), ρ_d_ is the density of the distillate solution (approximated as a constant of 998 kg m^–3^), and *A*_m_ is the active area of the membrane (40 cm^2^). Due to the absence of the external crystallizer (as depicted in Figure 1) in the experimental setup used in this study, vapor flux was expected to decrease during experimentation as carbonate minerals grow on the surface of the membrane and prohibit vapor transfer.

Each of the carbon mineralization experiments in this study was conducted using cocurrent circulation flow rates of 0.3 and 0.2 L min^–1^ for the feed and distillate solutions, respectively. A higher flow rate was used for the feed solution to impart a slight hydraulic pressure gradient in the direction of feed solution toward the distillate solution to aid in the immediate identification of pore wetting. The cross-flow velocities of the feed and distillate solutions were calculated to be 6.7 and 4.4 cm s^–1^, respectively, based on the channel geometry of the custom-made flow cell. For the trials in which sweeping gas membrane distillation (SGMD) was conducted, a stream of compressed dry air was directed through the flow cell (as opposed to chilled DI water used in the other trials) at a flow rate of 5 scfm. Each trial was conducted using these same operating parameters and conducted in three stages. First, the membrane was equilibrated using DI water heated to the target temperature for 1 h. Then, the baseline membrane flux and distillate conductivity were established using 100 mM NaCl feed solution for 2 h. This step enabled the identification of premature pore wetting that would indicate membrane defects. Following this step, the 100 mM NaCl feed solution was drained out of the feed channel/tubing, the system was rinsed with DI water, and then a 30 wt % MEA solution loaded with CO_2_ gas was introduced as the feed solution. MEA solution (30 wt %) was chosen due to the high capacity for CO_2_ storage at this composition as well as the common use of this composition of amine in industrial applications of flue gas capture.^86−90^ The experimental conditions for each trial are listed in Table 1.

**Table 1:** Experimental Conditions for Comparative Membrane Distillation–Crystallization Analysis

experiment label	**PVDF**	**PTFE**	**PVDF-Coco**	**Low temp.**	**SGMD**	**Low CO**_**2**_	**Mg**^**2+**^
membrane type	PVDF	PTFE	PVDF-Coco	PVDF	PVDF	PVDF	PVDF
MDC configuration	DCMD	DCMD	DCMD	DCMD	SGMD	DCMD	DCMD
feed temp. (°C)	50	50	50	40	50	50	50
metal (Ca^2+^ or Mg^2+^)	Ca^2+^	Ca^2+^	Ca^2+^	Ca^2+^	Ca^2+^	Ca^2+^	Mg^2+^
metal conc. (M)	0.18	0.18	0.18	0.18	0.18	0.18	0.18
CO_2_ saturation (%)	15	15	15	15	15	5	15
feed flowrate (L min^–1^)	0.3	0.3	0.3	0.3	0.3	0.3	0.3
distillate temp. (°C)	20	20	20	20		20	20
distillate flowrate (L min^–1^)	0.2	0.2	0.2	0.2		0.2	0.2
gas flowrate (scfm)					5		

## 3 Results and Discussion

### 3.1 Membrane Surface Properties

**Figure 3:**
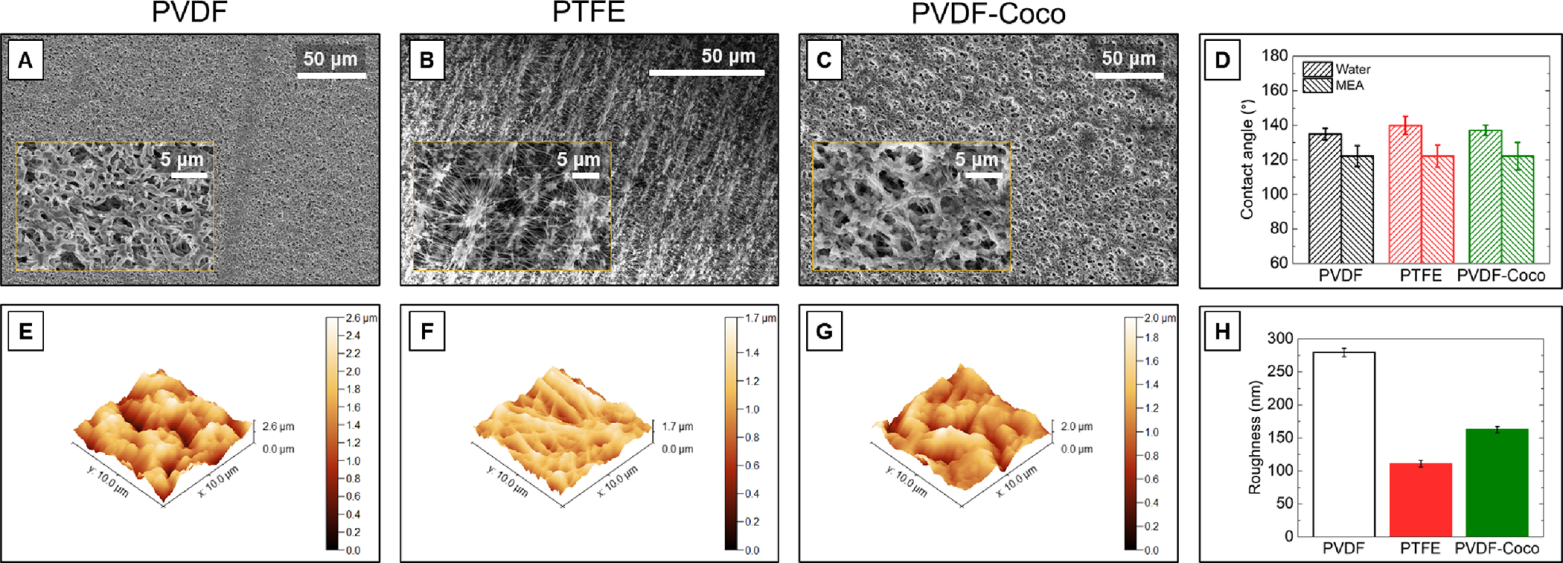
Scanning electron microscopy (SEM) images at different resolutions of the pristine (A) PVDF, (B) PTFE, and (C) PVDF-Coco membranes. (D) Static contact angles of each membrane using a 5 μL droplet of either deionized water or 30 wt % MEA solution. Atomic force microscopy (AFM) images of the active surface of the pristine (E) PVDF, (F) PTFE, and (G) PVDF-Coco membranes. (H) Arithmetic mean surface roughness calculated from the AFM data.

As membrane hydrophobicity, surface roughness, and chemical composition can independently affect the development of crystal nucleation propensity and morphologic variation,^91−94^ membrane properties were characterized prior to use. The pore structure, contact angle, and surface roughness for each of the three membranes tested (PVDF, PTFE, and PVDF-Coco) differed (Figure 3). The PVDF membrane chosen as the control material has a structure typical of polymeric membranes formed via phase inversion (Figure 3A). The PTFE membrane (of the same nominal pore size) exhibits a more stretched and fibrous structure that is indicative of the sintering and skiving procedure typically used in PTFE membrane fabrication (Figure 3B).^95^ The morphology of the PVDF-Coco membrane is nearly identical with that of the pristine PVDF membrane (Figure 3C) despite having lower surface roughness (Figure 3H). Plasma cleaning likely removed residual ambient microcontaminants that may have contributed to additional texture on the membrane surface;^96^ Huang et al. previously observed that surfaces similarly modified with naturally derived fatty acids remain unchanged physically.^97^ Each of the three membranes displays roughly the same static contact angle for both deionized water (140 ± 5°) and 30 wt % MEA (120 ± 4°) (Figure 3D). This contact angle range is in alignment with an average value of ∼135° from more than 1100 studies investigating PVDF and PTFE membrane surface properties in MD.^98^ For an ideal, smooth surface, the contact angle can be defined as the mechanical equilibrium of the drop under the action of three interfacial tensions (solid–vapor, solid–liquid, and liquid–vapor);^99^ however, surface topography also affects measured contact angles.^100,101^ In the context of MDC, crystal formation on membrane surfaces is favored on membranes with lower roughness and higher surface energy (i.e., low contact angle).^102−104^

Although PVDF is associated with higher surface energy compared to PTFE (Table S2), which would result in a lower contact angle in the absence of surface roughness, the greater surface roughness of the PVDF membrane (Figure 3H) likely increased the measured contact angle of the PVDF membrane (Figure 3D).^105−107^ The contact angle of the PVDF-Coco membrane is also similar to that of the pristine PVDF membrane despite the significantly lower surface energy of coconut oil (12.8 mN m^–1^). This is similarly likely due to the lower surface roughness of the PVDF-Coco membrane compared to the pristine PVDF membrane.

### 3.2 Carbon Mineralization Performance under Different Operating Conditions

In all experiments, MDC with transmembrane solvent flux and distillate conductivity monitoring was used to evaluate the effects of membrane surface properties and operating conditions on carbon mineralization. After the addition of alkaline earth metals (i.e., Ca^2+^ or Mg^2+^), the supersaturated crystallizing solution continued to flow over the membrane surface. *In situ* flux measurements were used to detect the development of crystal growth and subsequent pore blockage.

**Figure 4:**
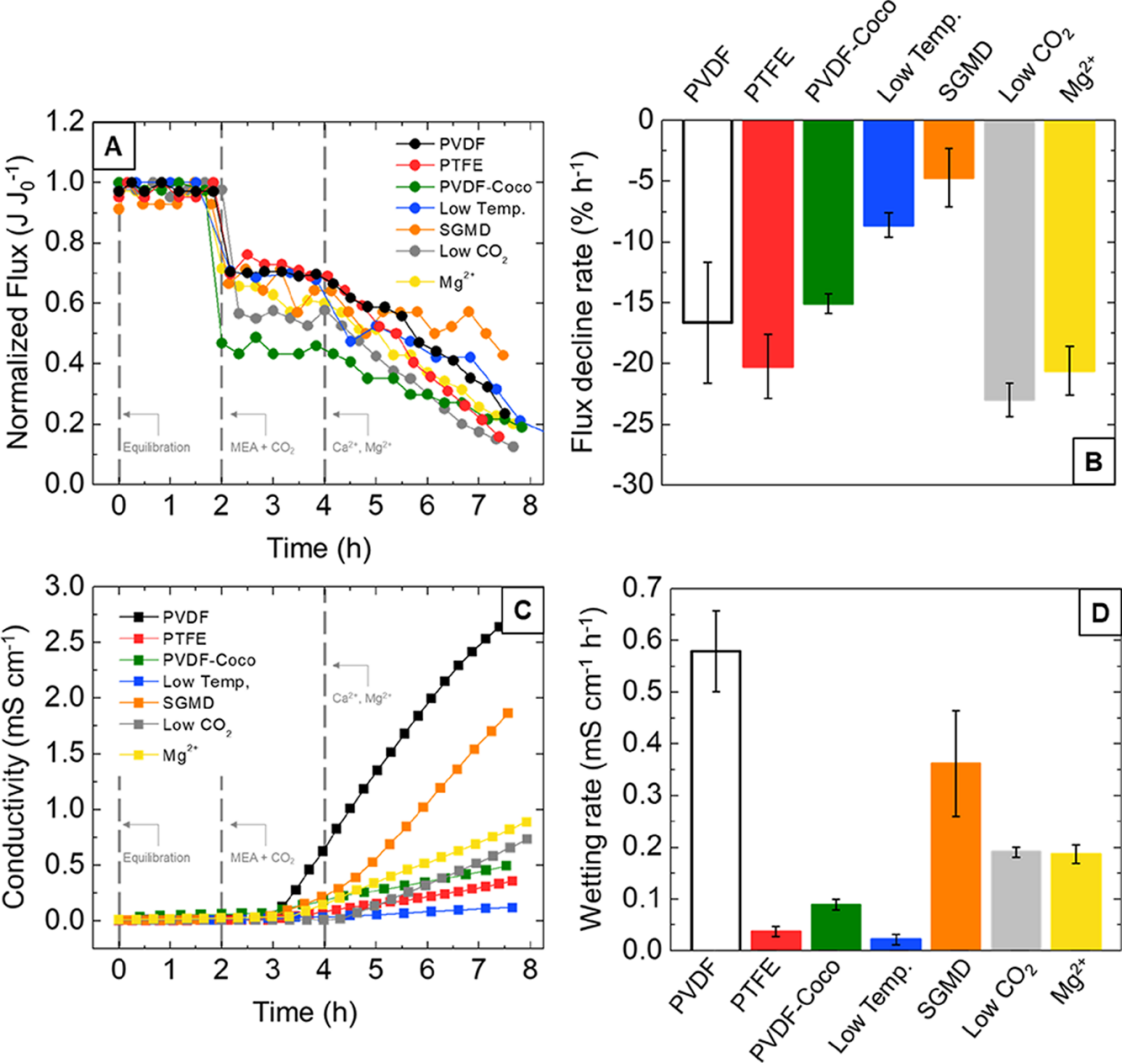
(A) Normalized flux over time, (B) flux decline rate, (C) distillate conductivity, and (D) wetting rate for each experimental condition tested. Experimental conditions are listed in Table 1. Each trial was run using 30 wt % monoethanolamine (MEA) solution mixed with 15% CO_2_ (or 5% CO_2_, in the case of the low CO_2_ trial) before being heated to 50 °C (or 40 °C, in the case of the low temperature trial) and dosed with Ca^2+^ (or Mg^2+^, in the case of the Mg^2+^ trial) to simulate alkaline earth metal wastes present in industrial residues. An equilibration solution was first circulated within the feed channel to establish a baseline flux, then the feed channel was drained and rinsed before MEA solution was circulated, and then, Ca^2+^ or Mg^2+^ was dosed to initiate carbon mineralization (gray dashed lines).

In all cases, normalized flux decreased upon the introduction of the CO_2_-loaded MEA feed solution at 2 h (Figure 4A) because its vapor pressure was lower than that of the equilibration solution.^108^ The average hourly flux decline rate (FDR) represents the change in transmembrane mass transfer over time, which here is primarily driven by crystalline species growing over and blocking membrane pores. Accordingly, FDR was used as the primary method for comparing the relative growth rates of the crystal species of interest (with a higher FDR representing more favorable crystal growth conditions). While scaling layer formation can be influenced by a number of factors (i.e., solution composition, pH, temperature, and concentration polarization within the membrane separation unit), scaling layer formation must be preceded by crystal nucleation and growth. Therefore, it is expected that the habit of crystal growth and nucleation can be extrapolated by interpreting the scaling layer formation via FDR. FDR is calculated based on the difference in flux after the introduction of Ca^2+^ or Mg^2+^ at 4 h.

Models have been developed to describe pore blockage in pressure-driven membrane separation.^109−111^ These models build upon Darcy-like relationships between transmembrane flux overall membrane resistance, which is composed of inherent membrane resistances as well as the resistances of the growing cake layer. This understanding of pore blockage is reflected in eq 1, where flux is inversely proportional to the active membrane area, which is participating in mass transfer (i.e., the portion of the membrane surface with open pores for vapor transport). In MDC, the loss of effective membrane area by crystal deposition has been confirmed using simultaneous quantification of air permeability through the membrane and the saturation level of the crystallizing species.^70^ Therefore, the FDR can be understood to represent the presence and temporal variation in the cake layer resistance on the membrane surface.

As shown in Figure 4B, each of the configurations tested fell within an FDR range of −22.9 to −4.71% h^–1^. The effects of membrane chemistry on FDR were dramatic, with the PTFE membrane exhibiting an FDR of −20.2% h^–1^ and the PVDF-Coco membrane exhibiting an FDR of −15.0% h^–1^, compared to the control configuration (PVDF), which exhibited an FDR of −16.6% h^–1^. From classical nucleation theory, it can be predicted that nucleating surfaces with the lowest surface energy present the highest energy barrier to nucleation for precipitating species.^112,113^ Therefore, it may be expected that the PTFE membrane, with a surface energy lower than that of the PVDF membrane, would display the lowest FDR due to a corresponding greater resistance to nucleation. However, the baseline flux through the PTFE membrane was more than 20% higher than that of the PVDF membrane (Table 2 and Figure S2). That is, the rate of pure water removal (or, equivalently, dewatering of the feed solution) was greater for the PTFE membrane, resulting in a more rapid availability of supersaturated ion conditions for mineral formation. Higher flux values lead to both faster arrival to conditions suitable for mineral nucleation and higher concentrations at the feed-membrane interface due to concentration polarization.^114^ It is notable that the difference in baseline flux displays a strong correlation to the difference in FDR for the PVDF and PTFE membranes. However, this trend is not reflected in the FDR of the PVDF-Coco membrane compared to that of the PVDF membrane. A higher baseline flux through the PVDF-Coco membrane correlated to a lower absolute FDR, potentially resulting from a lower precalcium MEA flux (Table 2). It is possible that the lower MEA flux observed through the PVDF-Coco membrane is due to a partial displacement of the fatty acid coating into the membrane pores as the MEA solution was introduced to the system. It is notable that the highest values for roughness and surface energy are observed for the PVDF membrane, yet the PVDF membrane displayed a smaller FDR compared with the PTFE membrane. With more abundant nucleation sites afforded by increased roughness and a lower energy barrier for nucleation due to lower hydrophobicity, the PVDF membrane was poised, in theory, to rapidly facilitate crystallization, which would be expected to result in larger FDR. However, the lower baseline flux of the PVDF membrane, as compared to that of the PTFE membrane, empirically seems to have been a more influential factor for FDR than roughness or surface energy.

**Table 2:** Experimental Results for Water Flux, monoethanolamine (MEA) Solution Flux, Flux Decline Rate, and Wetting Rate for Each of the Trials Conducted under Varying Carbon Mineralization Conditions

experiment label	**PVDF**	**PTFE**	**PVDF-Coco**	**low temp.**	**SGMD**	**low CO**_**2**_	**Mg**^**2+**^
membrane type	PVDF	PTFE	PVDF-Coco	PVDF	PVDF	PVDF	PVDF
measured water flux (L m^–2^ h^–1^), 0–2 h	25.1 ± 1.5	30.8 ± 1.8	27.4 ± 1.6	14.1 ± 0.8	9.94 ± 0.6	28.9 ± 1.7	24.1 ± 1.4
measured mean flux (L m^–2^ h^–1^), 2–4 h	17.9 ± 1.1	23.0 ± 1.4	12.6 ± 0.8	10.0 ± 0.6	6.84 ± 0.4	16.0 ± 1.0	16.9 ± 1.0
measured flux decline rate (L m^–2^ h^–2^), 4–8 h	–2.74 ± 0.8	–4.55 ± 0.6	–1.88 ± 0.1	–0.86 ± 0.1	–0.47 ± 0.2	–3.33 ± 0.1	–2.88 ± 0.3
normalized flux decline rate (% h^–1^), 4–8 h	–16.6 ± 4.9	–20.2 ± 2.62	–15.0 ± 0.8	–8.58 ± 1.0	–4.71 ± 2.4	–23.0 ± 1.4	–20.6 ± 2.0
measured wetting rate (mS cm^–1^ h^–1^), 4–8 h	0.58 ± 0.1	0.04 ± 0.01	0.09 ± 0.01	0.02 ± 0.01	0.36 ± 0.1	0.19 ± 0.01	0.19 ± 0.02

All membranes exhibited pore wetting. The primary mechanism for pore wetting in carbon mineralization is expected to be physical pore deformation and reduction of liquid entry pressure as crystals grow within pore openings.^75,98,115^ When membrane wetting occurs, the dissolved ions within the feed solution penetrate into membrane pores, which results in a notable increase in distillate electrical conductivity.^116^ The average hourly wetting rate (WR) was quantified as the change in distillate conductivity over time. A WR of 0.58 mS cm^–1^ h^–1^ was observed for the PVDF membrane (Table 2). This WR was the highest of any trial conducted and nearly 6 times higher than either the PTFE or PVDF-Coco membranes, which displayed WR values of 0.04 and 0.09 mS cm^–1^ h^–1^, respectively. These results indicate that the PVDF-Coco membrane successfully delayed membrane wetting. The PVDF-Coco membrane enabled mineral generation with a lower propensity for pore wetting than the PVDF membrane but required a slightly longer time to generate the minerals in the first place. The PTFE membrane enabled faster mineral production as well as less pore wetting when compared to the PVDF and PVDF-Coco membranes.

**Figure 5:**
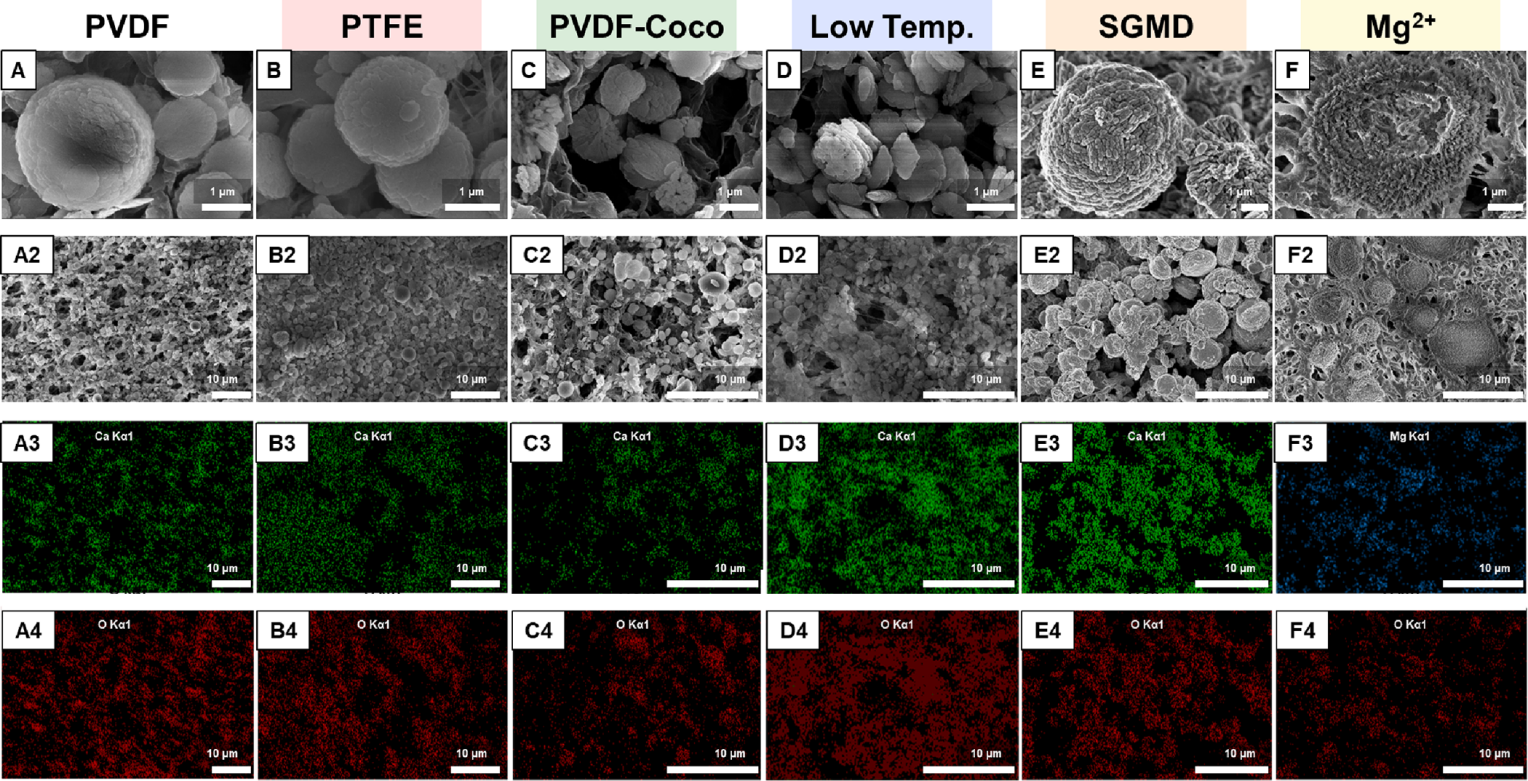
(A–F) Scanning electron microscopy with elemental dispersive X-ray spectroscopy analysis (SEM-EDX) of the surfaces of the tested membranes and configurations. X-ray mapping of the (A3–F3) calcium and (A4–F4) oxygen species. Electron microscopy was conducted without applying any cleaning procedure beforehand.

Carbonate mineral morphology is relevant to MDC because the commercial products made with carbonates (such as coarse aggregate or concrete) typically must meet ASTM standards to ensure safety and durability.^117−119^ No distinct differences were observed in crystal morphology across the membranes and conditions tested (Figure 5A–C). All membranes and conditions tested yielded spherical particles with a diameter of ∼2 μm and a chemical identity strongly represented by Ca and O according to EDX analysis (Figure 5A3–C3). Additionally, XRD analysis shows distinct agreement between the crystals harvested from experimentation and the standard card for the calcite (not aragonite or vaterite) polymorph of calcium carbonate (Figure S3). Separately, the abundance of crystals on the membrane surface, as quantified using ImageJ to determine the percentage of crystal coverage within a unit area, correlated closely with membrane surface energy (Figures S4 and S5).^120^ The highest crystal density was observed on the PVDF membrane with 74.1% coverage followed by PTFE and PVDF-Coco membranes with 60.3 and 48.0% coverage, respectively.

In addition to exploring the influence of membrane characteristics, operating parameters were also investigated to explore their influence on carbon mineralization. The operating parameters investigated were feed solution temperature (50 vs 40 °C), MD configuration (DCMD vs SGMD), CO_2_ loading (15 vs 5%), and cation identity (Ca^2+^ vs Mg^2+^).

Temperature dramatically affects both crystal growth kinetics and crystal solubility (Figure S6).^121,122^ A modest decrease in the process temperature from 50 to 40 °C was imposed to simulate differences in thermal energy available from different industrial sources.^123^ As expected, a lower baseline flux of 10.0 LMH was observed for the 40 °C trial compared to 17.9 LMH for the 50 °C control trial (Figure S2 and Table S1). Although the FDR observed during the 40 °C trial was far lower than that of the 50 °C trial, with values of −8.58 and −16.6% h^–1^, respectively, the WR was far more tolerable, with values of 0.0221 and 0.579 mS cm^–1^ h^–1^, respectively. The reduced FDR at lower feed temperature is likely due to the differences in calcite solubility (i.e., less chemical driving force for nucleation at lower temperature) as well as differences in the concentration at the feed-membrane interface (i.e., fewer ions present in the zone of crystallization) due to concentration polarization effects. These results agree with previous studies in which lower feed temperature both elongates mineral generation time and reduces WR.^124,125^

The lowest FDR of −4.71% h^–1^ was observed for the SGMD configuration, with a corresponding WR of 0.36 mS cm^–1^ h^–1^. While this WR was lower than that of the control direct contact membrane distillation (DCMD) trial (0.58 mS cm^–1^ h^–1^), it is higher than WR values observed under all other noncontrol experimental conditions, which may be a result of the higher interfacial feed temperature (resulting in reduced surface tension of the feed solution at pore entrances^126^) or sweep gas partially swelling the membrane pores (due to Bernoulli’s principle) and facilitating wetting. Pore swelling can relax the internal stresses within the interconnected membrane matrix to decrease the membrane’s liquid entry pressure and cause premature feed vapor condensation within the pores,^125^ thereby enabling a faster liquid linkage between the feed and distillate channels on either side of the membrane. While colder sweep gas temperature and higher sweep gas flow rate are correlated with higher vapor flux across the membrane, the smallest baseline flux was observed in the SGMD configuration due to the ambient temperature of the sweep gas and the relatively low flow rate of the sweep gas (173 g min^–1^ or 5 scfm) compared to the feed solution (300 g min^–1^ or 0.3 L min^–1^).

The effect of CO_2_ loading on carbon mineralization was also investigated to determine whether variation in available carbonate ions (eqs S4–S7) would influence the generation of crystals during MDC. The similarity in FDR between the control condition and the low CO_2_ condition (16.6 and 23.0% h^–1^, respectively) suggests that the CO_2_ concentration has little effect on the growth rate of crystals within the range of conditions explored in this study. Previous studies on carbonate crystallization have indicated that lower CO_2_ concentrations impart two opposing effects simultaneously: (i) fewer carbonate ions present in the feed environment leading to more sluggish mineral growth and (ii) less dissolved CO_2_ in the feed solution resulting in a higher starting pH value (due to the acidifying effect of CO_2_ dissolution in solution), which would lead to more aggressive mineral growth.^127^ A low stoichiometric quantity of available carbonate ions would serve as a limiting reactant to potentially serve as a tuning knob to control the size of the calcite crystals. At higher pH values, calcite is more readily precipitable from solution (Figure S7). The low CO_2_ condition enabled a higher pH value by restricting the presence of carbonic acid in the solution (CO_2_ reacts with water to form carbonic acid, CO_2_ + H_2_O → H_2_CO_3_).^128^ The observation that the low CO_2_ condition displayed a slightly faster FDR than the control condition can be explained by either a higher baseline MEA flux (i.e., more rapid concentration of the feed solution and thus shorter crystal induction time) or a lower tendency to form crystals at higher pH. Because both CO_2_ loadings resulted in a similar measured baseline MEA flux (Figure S2), it is likely that the pH effects were more influential than the effects of the varied CO_2_ concentration.

Magnesium (Mg^2+^) is highly influential in the morphology and growth of calcite in natural systems.^129^ Calcite growth is delayed in the presence of Mg^2+^ ions, possibly due to changes in calcite solubility when Mg^2+^ is incorporated as an impurity during a step-pinning process where the rate of crystal edge growth is reduced.^130^ For the proposed system, mixed precursor ion samples from desalination brine, cement kiln dust, steel slag, and other ash-producing or brine-producing industrial processes pose interesting possibilities for indirectly tuning crystal generation. While competitive and synergistic effects of mixed Mg^2+^ and Ca^2+^ solutions are of keen interest, the singular ions were explored in this study to identify any differences in the crystal growth rate or pore wetting individually. The baseline flux and FDR for the Mg^2+^ condition and the Ca^2+^ control condition were similar (4.1% difference for baseline flux and 4.9% difference for FDR), but the WR for the Mg^2+^ condition was more than 3 times lower (0.58 vs 0.19 mS cm^–1^ h^–1^). This was likely because the solid crystalline transitional phase of MgCO_3_ exhibits resistance to shear stress, while the amorphous noncrystalline transitional phase of CaCO_3_ does not.^131−133^ Therefore, the early stage molecules of CaCO_3_ are likely more easily compressed into the mouth of the membrane pores than the early stage molecules of MgCO_3_, leading to a longer membrane lifetime during carbon mineralization with Mg^2+^. Such an observation may inspire future process optimization routes in which magnesium carbonates are selectively crystallized before calcium carbonates to mitigate pore wetting in mixed solutions.

Scaling is expected to be the most significant mechanism for flux reduction,^134,135^ but it remains to be understood whether scaling reduces flux directly or indirectly (or both). Indirect flux decline can be caused when a temperature-insulating crystal layer grows around the membrane’s pores (i.e., not within the mouth of the pores), thereby causing temperature polarization at the feed-membrane interface before a significant percentage of membrane pores becomes directly blocked by the crystals.^65,136^ Within the temperature range relevant for MDC (40–90 °C), carbonate minerals have thermal conductivity values ranging from 2 to 5 W m^–1^ K^–1^,^137,138^ depending on species and porosity, while polymers typically used in MDC (i.e., PVDF, PTFE, and PP) have thermal conductivity values ranging from 0.01 to 0.3 W m^–1^ K^–1^.^139,140^ This 1–2 orders of magnitude difference between the thermal conductivity of the crystal layer and the membrane may play a synergistic role with the pore blockage phenomenon to fully explain the flux decline in MDC. Whether the influence is direct or indirect, the occurrence of flux decline during the MDC process is an informative tool for the comparative analysis of crystal growth. Furthermore, the flux decline rate gives an intuitive understanding of how quickly crystals develop on the membrane surface under each experimental condition, thereby shedding light onto practical considerations of carbon mineralization.

### 3.3 Feasibility Considerations

Reconciling the environmental, social, and financial considerations has remained a highly controversial issue for carbon capture, utilization, and storage (CCUS) technologies. The global generation rate for alkaline ashes is greater than 1 Gt year^–1^,^141,142^ which, if used completely for carbon mineralization, could offset CO_2_ emissions by more than 4.02 Gt CO_2_ year^–1^ (roughly 10.9% of the 36.8 Gt CO_2_ global emissions total in 2022).^143,144^ MDC for alkaline amine looping has the potential to provide reliable, controllable carbon utilization and storage, but several factors must be considered. The process must be integrated with the production of both alkaline ashes and CO_2_ while also being collocated with effective sources of waste heat. Also, locations for carbon mineralization should primarily be near markets that heavily value calcium or magnesium carbonates, such as construction supply factories, pulp and paper processing facilities, and paint manufacturing facilities.

**Figure 6:**
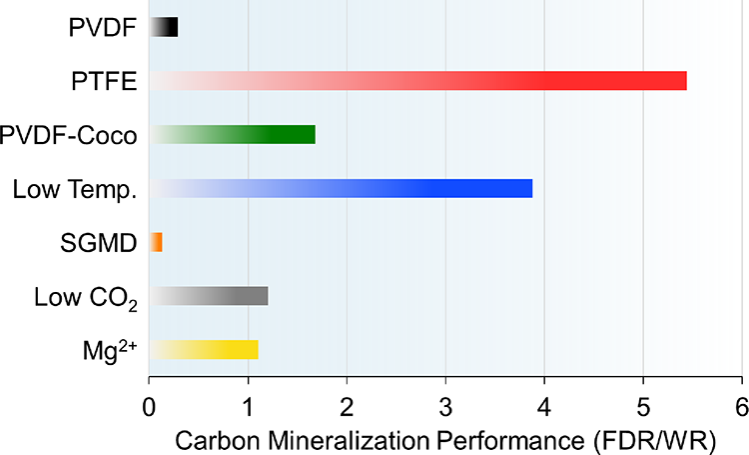
Quotient of the absolute value of the flux decline rate (FDR) and the wetting rate (WR) calculated for each of the conditions tested. This metric represents the relative carbon mineralization performance with greater FDR/WR being desirable. By comparing the combined dependent variables, the relative influence of each separate independent variable tested is directly observable.

The feasibility of CO_2_ mineralization using MDC for amine looping is a function of many factors. After identifying suitable locations that satisfy the above conditions, the extent and timing of the carbonation reactions must be determined. This involves the identification of the reaction kinetics for different minerals as well as the effects of the variables explored here. Then, it is necessary to discuss the environmental effects and economic constraints of implementing such a process at scale. The relative extent of carbonation and precipitation reaction kinetics can be approximated indirectly by using FDR, and the robustness of the membrane employed can be evaluated by using WR. Larger FDR represents more rapid generation of calcite on the membrane surface, while smaller WR represents slower membrane failure. Therefore, the ratio of FDR to WR can be understood to represent relative carbon mineralization performance, with greater FDR/WR being desirable (Figure 6).

Higher values for FDR/WR are achieved with a large FDR and/or small WR. The greatest carbon mineralization performance was observed for the PTFE membrane with an FDR/WR value of 5.4, and the lowest was for the PVDF membrane under the SGMD configuration with 0.13, indicating that future efforts should be directed toward approaches that can offer higher baseline flux and mineral production with minimal wetting. However, the high FDR/WR for the 40 °C condition (low temperature) indicates that higher temperatures may not be the best route for achieving high baseline flux. Rather, material selection and, perhaps, other configurations, such as vacuum MD, should be prioritized to maximize flux without increasing temperature. The PVDF-Coco membrane displayed improved performance compared to the PVDF control condition with FDR/WR values of 1.7 and 0.29, respectively, demonstrating that modifications of membrane chemistry can provide a marginal benefit but are potentially less impactful than temperature adjustments. The trade-off, however, is that the nominal crystal generation rate will be lower for a lower temperature condition with all other parameters being equal. The conditions of low CO_2_ loading and Mg^2+^ carbonation both displayed higher FDR/WR values than the control condition and should be explored in future studies with varying membrane chemistries and mixed conditions for further optimization.

## 4 Implications

This study demonstrates a proof-of-concept study and evaluation of the application of low-grade temperature-driven MDC to recover CO_2_ in the form of valuable carbonate minerals from synthetic postcombustion flue gas scrubbing solutions. This method allows for the utilization of flue gas scrubbing wastewater and CO_2_ to produce valuable materials while also enabling the continued use of absorbing solvents without excessive energy inputs.

It was found that membranes with lower surface energy and lower roughness displayed mineral production rates that were ∼25% higher and wetting rates that were ∼94% lower compared to a control membrane. Employing a lower temperature for vapor pressure control reduced mineral production rates by ∼48% and also reduced membrane wetting by more than 95%. Interestingly, reducing CO_2_ concentrations resulted in similar or higher mineral production rates and reduced wetting by ∼67%. Crystal morphology was consistent within the range of operating parameters studied. Although this work has demonstrated the promise of MDC for carbon mineralization, further work is needed to evaluate mineral production and membrane health when it is coupled with a brine crystallizer.

Compared with existing approaches for managing flue gas scrubbing wastewater, our method offers a novel synergistically regenerative alternative that maximizes the use of otherwise wasted products (i.e., minerals, solvents, and convective heat). This work provides a foundation for the development of novel circular-resource-enabling CCUS technologies to commercially feasible levels.

## Supporting Information Available

Additional experimental details, material properties, and reference data (PDF)
