# Supplementary material for: Membrane Distillation–Crystallization for Sustainable Carbon Utilization and Storage
Source: Environ Sci Technol. 2023 Oct 19;57(43):16628–40. doi: 10.1021/acs.est.3c04450 (PMC10621001; doi:10.1021/acs.est.3c04450)
Supplement: Supplementary file 1 — es3c04450_si_001.pdf [file es3c04450_si_001.pdf]

Supporting Information for

Membrane Distillation-Crystallization for Sustainable Carbon Utilization and Storage

Kofi S.S. Christie[1,2*], Allyson McGaughey[1,3], Samantha A. McBride[4], Xiaohui Xu[3], Rodney D. Priestley[3,5], Zhiyong Jason Ren[1,2*]

[1]Andlinger Center for Energy and the Environment, Princeton University, Princeton, New Jersey 08544, United States

[2]Department of Civil and Environmental Engineering, Princeton University, Princeton, New Jersey 08544, United States

[3]Department of Chemical and Biological Engineering, Princeton University, Princeton, New Jersey 08544, United States

[4]Department of Mechanical and Aerospace Engineering, Princeton University, Princeton, New Jersey 08544, United States

[5]Princeton Institute for the Science and Technology of Materials, Princeton University, Princeton, New Jersey 08544, United States

*Corresponding authors: kchristie@lsu.edu; Tel: +1 (225) 578-1523
zjren@princeton.edu; Tel: +1 (609) 258-7580

Contents:

13 Pages

Supporting Text

2 Supporting Tables

9 Supporting Figures

Supporting Text

Chemistry of carbon mineralization

The chemical equilibrium reactions for the mineralization of each of ideal candidate species (i.e., wollastonite ($CaSiO_3$), olivine (Mg_2SiO_4), and serpentine ($Mg_3(OH)_4Si_2O_5$) are shown in **Equation S1** – **Equation S3**, respectively.[1] Similar mineralization pathway reactions exist for other species of interest (i.e., anorthite, augite, anorthosite, basalt).[2]

$$CaSiO_{3(s)} + CO_{2(g)} \rightarrow CaCO_{3(s)} + SiO_{2(s)}$$ **Equation S1**

(Wollastonite, 90 kJ/mol CO_2 released)

$$Mg_2SiO_{4(s)} + 2CO_{2(g)} \rightarrow 2MgCO_{3(s)} + SiO_{2(s)}$$ **Equation S2**

(Olivine, 89 kJ/mol CO_2 released)

$$Mg_3Si_2O_5(OH)_{4(s)} + 3CO_{2(g)} \rightarrow 3MgCO_{3(s)} + 2SiO_{2(s)} + 2H_2O_{(l)}$$ **Equation S3**

(Serpentine, 64 kJ/mol CO_2 released)

In each of the above natural reactions, the weathering of the mineral species is promoted by the dissolution and subsequent ionization of CO_2 in the rain or groundwater, which yields carbonic acid (H_2CO_3). Then, the H_2CO_3 dissociates to form protons (H^+) and bicarbonate (HCO_3^-). Because HCO_3^- is a conjugate acid, it can chemically degrade target minerals, liberate Ca^{2+} and Mg^{2+}, and enable their reaction with bicarbonate to form solids from aqueous solutions.

In systems where pure CO_2 is not available, another chemical reaction must precede the formation of HCO_3^- for mineralization; that is, CO_2 gas must be separated from the carbamate molecules that form within the aqueous amine during carbon capture. These reactions are summarized as follows: first, gaseous CO_2 is diffused into the amine solution, which results in the formation of the zwitterionic intermediate (**Equation S4**). The intermediate is then further reacted in the amine solution to form stable carbamate (**Equation S5**). Then, upon heating, the carbamate can readily be converted back into both the original amine for further CO_2 capture, and into the CO_3^{2-} ions that form carbonate minerals (**Equation S6** and **Equation S7**).[3]

$$CO_2 + RNH_2 \rightarrow RNH_2^+CO_2^-$$ **Equation S4**

$$RNH_2^+CO_2^- + RNH_2 \rightarrow RNH_3^+ + RNHCO_2^-$$ **Equation S5**

$$H_2O + RNHCO_2^- \rightarrow RNH_2 + HCO_3^-$$ **Equation S6**

$$HCO_3^- \rightarrow H^+ + CO_3^{2-}$$ **Equation S7**

R represents CH_2CH_2OH for the MEA molecule.

Membrane selection

The process similarities between membrane distillation-crystallization (MDC) and membrane distillation (MD), the surface energy of the membrane polymer must be as low as possible to avoid pore wetting.[4] Pore wetting is the infiltration of liquid into the pore space of the membrane, which compromises water-salt selectivity.[5] High surface tension liquids such as water are the typical target for recovery from feed solutions, and the membranes suitable for MD have traditionally been very hydrophobic, low surface energy materials such as polytetrafluoroethylene, poly(vinylidene fluoride), polyethylene, or polypropylene.[4] Many studies have identified surface modification procedures to further reduce the surface energy of these membranes with materials such as TiO_2 nanoparticles,[6] vapor-phase silanized silica nanoparticles,[7] and naturally-derived fatty acids.[8,9] While vapor transport is primarily governed by the vapor-liquid equilibrium at the pore-feed interface (and thus cannot be optimized by material properties), flux can be optimized by selecting a membrane with sufficient porosity. Higher porosity is typically afforded by larger pore size, but larger pores are more susceptible to pore wetting.[4] Membranes with a high porosity (70 to 80%) with pore sizes around 0.2 to 0.45 μm are desirable.[10] Additionally, a narrow pore size distribution reduces the risk of wetting because the largest pores will be the first to wet, as deduced by Kim and Harriot in a system of equations (**Equation S8** and **Equation S9**) similar to the Young-Laplace equation.[11,12]

$$\Delta P = -\frac{2\gamma}{r}\frac{\cos(\theta - \alpha)}{1 + \frac{R}{r}(1 - \cos\alpha)}$$ Equation S8

$$\sin(\theta - \alpha) = \frac{\sin\theta}{1 + r/R}$$ Equation S9

Where ΔP is the hydraulic pressure between the liquid side and the gas side of a membrane pore, γ is the surface tension, θ is the contact angle measured from inside the liquid phase, α is the critical angle calculated to compensate for noncylindrical pore geometry, r is the apparent pore radius, and R is the radius of the membrane fiber.

Regarding membrane thickness, higher vapor flux and higher thermal efficiency in MD are generally afforded by using thin and thermally insulating membranes.[13] Scaling, or the buildup of mineral precipitates on a membrane surface under supersaturated feed conditions, is an occurrence that requires careful attention in MD. In order to optimize scaling resistance and flux recovery via membrane cleaning, membranes should have a high degree of roughness and a low surface energy, which are often afforded by coating the membrane with nanoparticles grafted with perfluorinated functional groups or other ultralow surface energy molecules (although many other techniques can be used).[14,15]

Polyvinylidene fluoride (PVDF) membranes were selected as the control membrane material due to the wide usage of PVDF in previous studies on membrane distillation crystallization.[16–18] The wide usage of PVDF is usually attributed to the high permeate flux and mechanical robustness afforded by PVDF membranes.[19] The PTFE membrane was selected for comparison because it is also a commonly used

membrane material in MD and MDC. Also, PTFE exhibits a more stretched and fibrous structure with a similar surface energy to PVDF, thereby enabling the extraction of any insights related to the internal pore structure of commercially available membranes. The PVDF-Coco membrane was selected for comparison because it demonstrates the use of naturally-derived fatty acids to modify the commercial membrane surface and offer enhanced performance without the use of additional fluorinated material, which carries negative environmental implications.

Fluoropolymer materials are generally preferred for membrane applications in which the inner pore space must remain dry, due to their high hydrophobicity. However, the manufacturing process of fluoropolymers historically involved use of perfluorooctanoic acid (PFOA) and perfluorononanoic acid (PFNA), and currently typically involves use of GenX, an alternative to PFOA. Perfluoroalkyl substances including PFOA and PFNA are associated with various health effects observed in animal models and humans.[20–22] GenX was recently added to the EU Candidate List of Substances of Very High Concern due to its similarities to other PFAS in environmental persistence, mobility, and observed health effects.[23] Precursor chemicals such as 1H,1H,2H,2H-perfluorodecyltriethoxysilane (FAS) are widely used for the fabrication of low surface energy materials due to the low interfacial tension (18.1 mN m^{-1}) of the chemical. In general, it would be environmentally beneficial to avoid the use of perfluorinated compounds, and to instead pursue the development of plant-based alternatives, such as coconut oil-derived fatty acids which can enable comparable or even lower surface energy (12.8 mN m^{-1}) than fluorinated compounds (**Table S2**).

Supporting Tables

Table S1 – Technological readiness level of four promising carbon dioxide management strategies.

Technology Pathway	Potential Products	Attributes	Technological Readiness Level
Thermochemical	Chemicals, materials, and fuels	Can use abundant natural gas in the United States	2-5
Electrochemical and photochemical	Chemicals, materials, and fuels	Can use the excess renewable energy	1-4
Carbon mineralization	Construction materials, specialty materials (e.g., fillers)	Offers great permanency	5-9
Biological conversion	Chemicals and fuels	Permanency depends on product; slower inherent kinetics than alternative use pathways	3-9

Table S2 – Surface energies or interfacial tensions of typical MDC polymeric compounds and surface-modifying molecules

Material	Surface energy or interfacial tension (mN m^{-1})	Reference
Polyvinylidene fluoride (PVDF)*	33	[24]
Polypropylene-isotactic (PP)	34	[24]
Polytetrafluoroethylene (PTFE)*	22	[24]
1H,1H,2H,2H-perfluorodecyltriethoxysilane (FAS)	18.1	[25]
Coconut oil*	12.8	[26]

*Used in this study

Supporting Figures

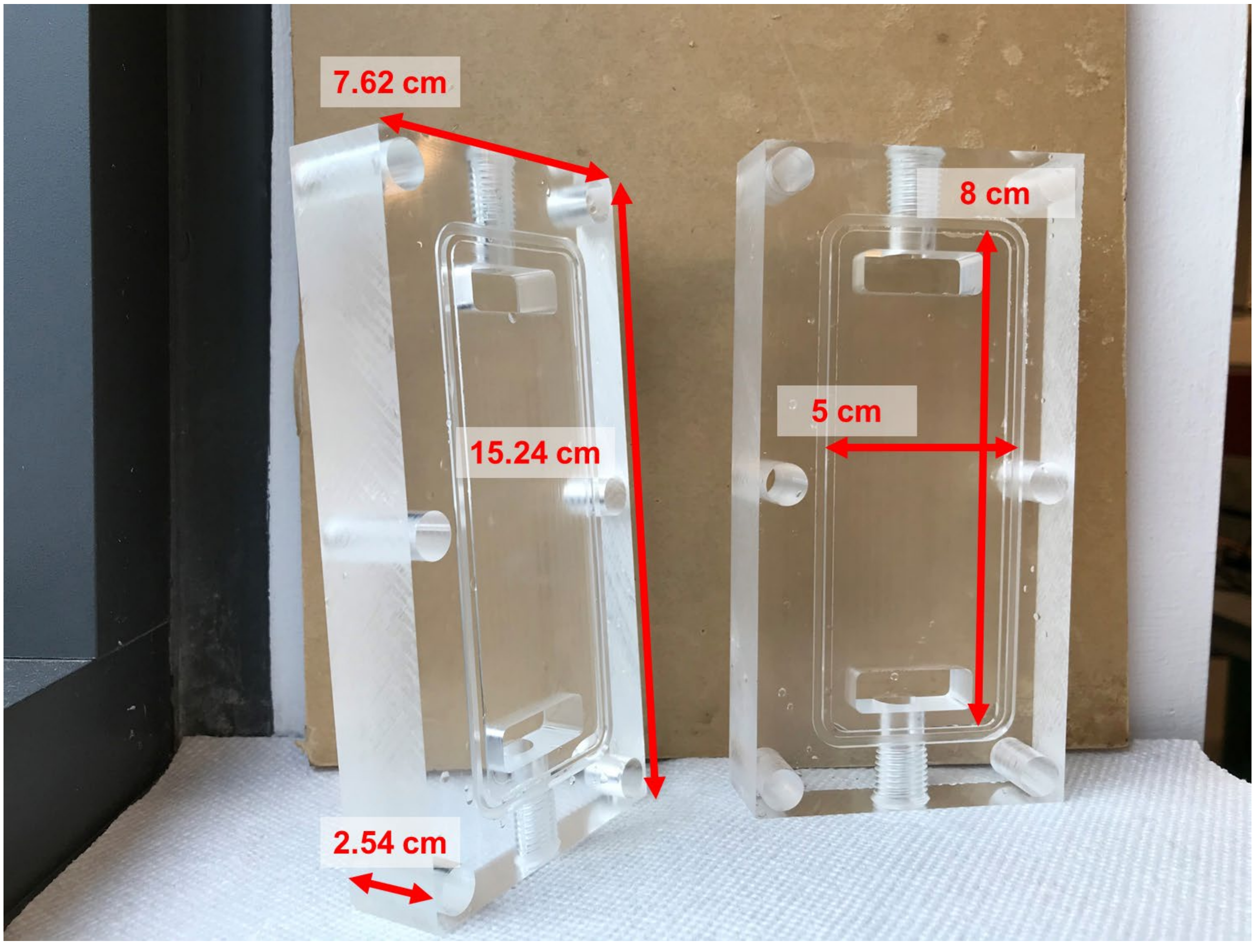

Figure S1 – Dimensions of the custom-built membrane test cell plates. The identical inlet and outlet ports on each half-shell enable consistent flow patterns through the rectangular inlet and outlet channels regardless of orientation. The six circular through-holes on each half-shell are holes through which bolts are inserted for precise sealing while avoiding any creasing or wrinkling of the membrane coupon. The channel surrounding the active area of each half-shell accommodates a chemically resistant buna-n rubber o-ring for leak-proof operation. Each channel can accommodate both liquid flow and air flow to enable varying membrane distillation-crystallization (MDC) configurations. Although hollow fiber membranes are more common in industrial and commercial applications due to a larger active area for MD separation than flat sheet membranes, flat sheet membranes were used in this study because they are more easily autopsied and more reproducibly fabricated than hollow fiber membranes.[86]

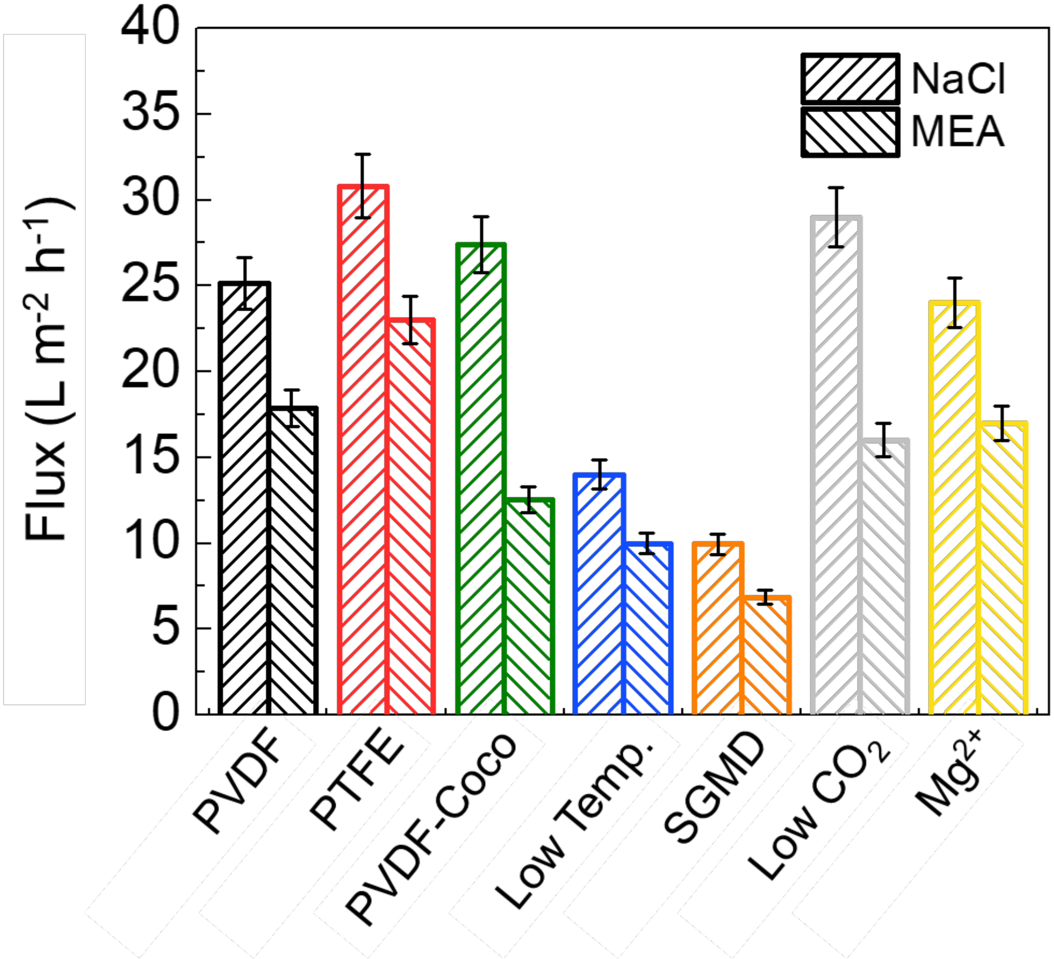

Figure S2 – Transmembrane solvent flux for each experimental condition for 1) an equilibrating feed solution of 100 mM NaCl and 2) the MEA- and CO_2-loaded feed solution for carbon mineralization.

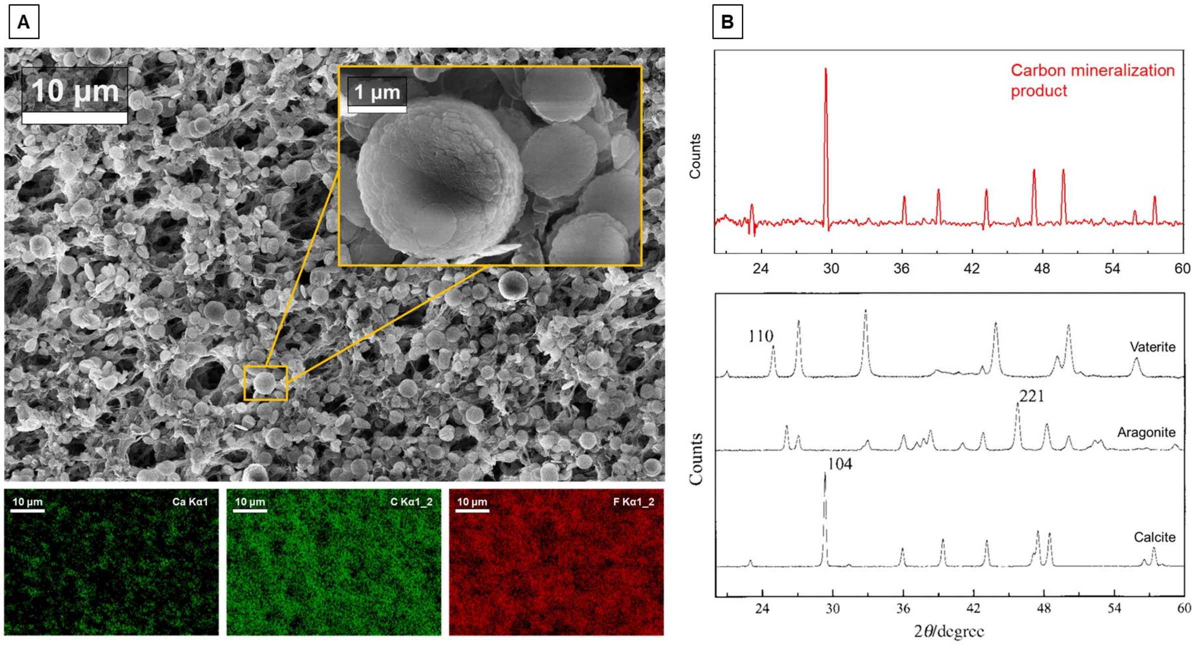

Figure S3 – (A) Scanning electron microscopy (SEM) images and corresponding energy-dispersive X-ray (EDX) analysis, as well as (B) X-ray diffraction analysis of the carbon mineralization product formed on the membrane surface.

Gibbs Free Energy Barrier for Nucleation:

$$\Delta G_{het}^{*} = \frac{16\pi\gamma_{ln}^{3}\Omega^{2}}{3(k_B T\sigma)^{2}}\left[\frac{1}{4}(1-\cos\theta)^{2}(2+\cos\theta)\right]$$

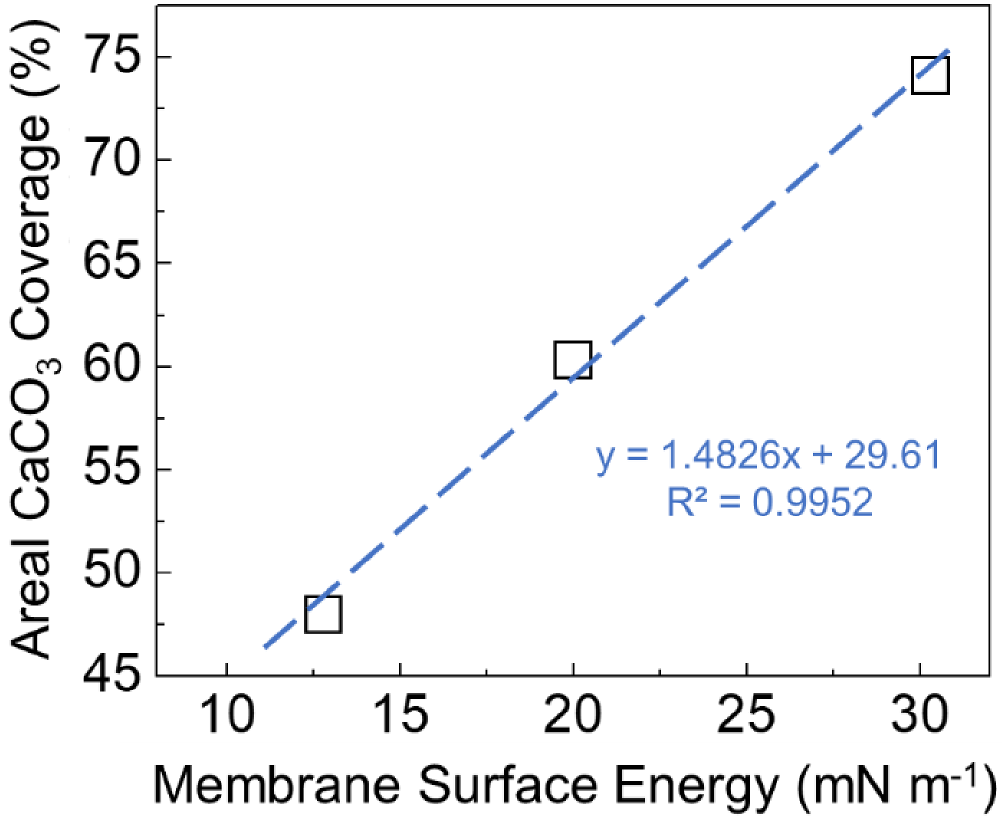

Figure S4 – Areal coverage of calcium carbonate ($CaCO_3$) vs membrane surface energy. The Gibbs free energy barrier for nucleation is positively correlated with the intrinsic contact angle between a nucleus and the substrate it adheres to (when both are submerged in solution) between π/2 and π radians. This contact angle is itself correlated with membrane sessile drop water contact angle.

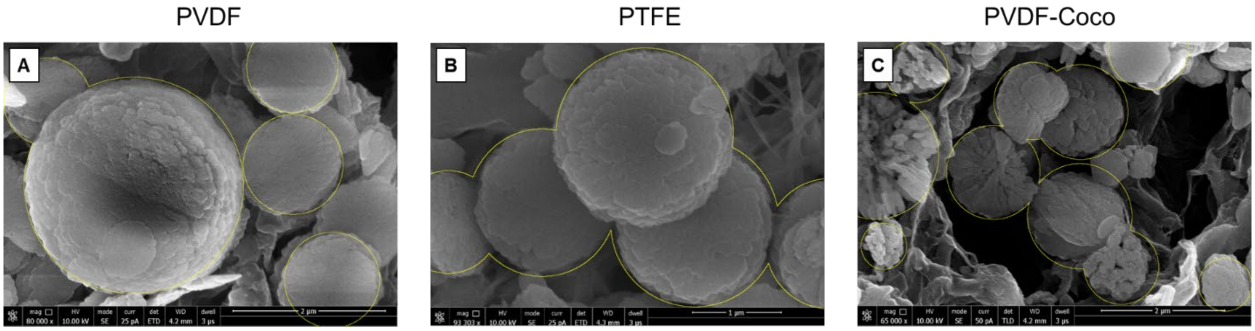

Figure S5 – Representative images of crystallized membrane scanning electron micrographs used for determination of areal coverage of calcium carbonate for the (A) PVDF, (B) PTFE, and (C) PVDF-Coco membranes used in experimental trials.

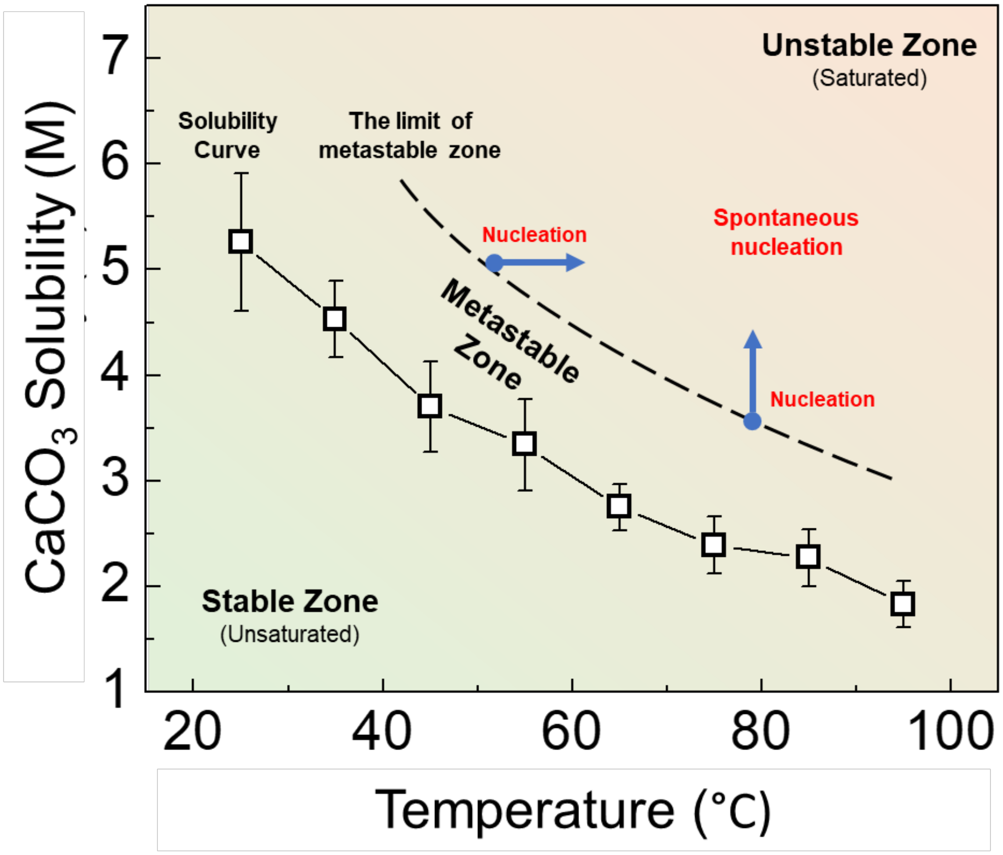

Figure S6 – Solubility vs. temperature plot for calcium carbonate ($CaCO_3$). The white squares represent measured solubility data from Coto et al. (2012).[27]

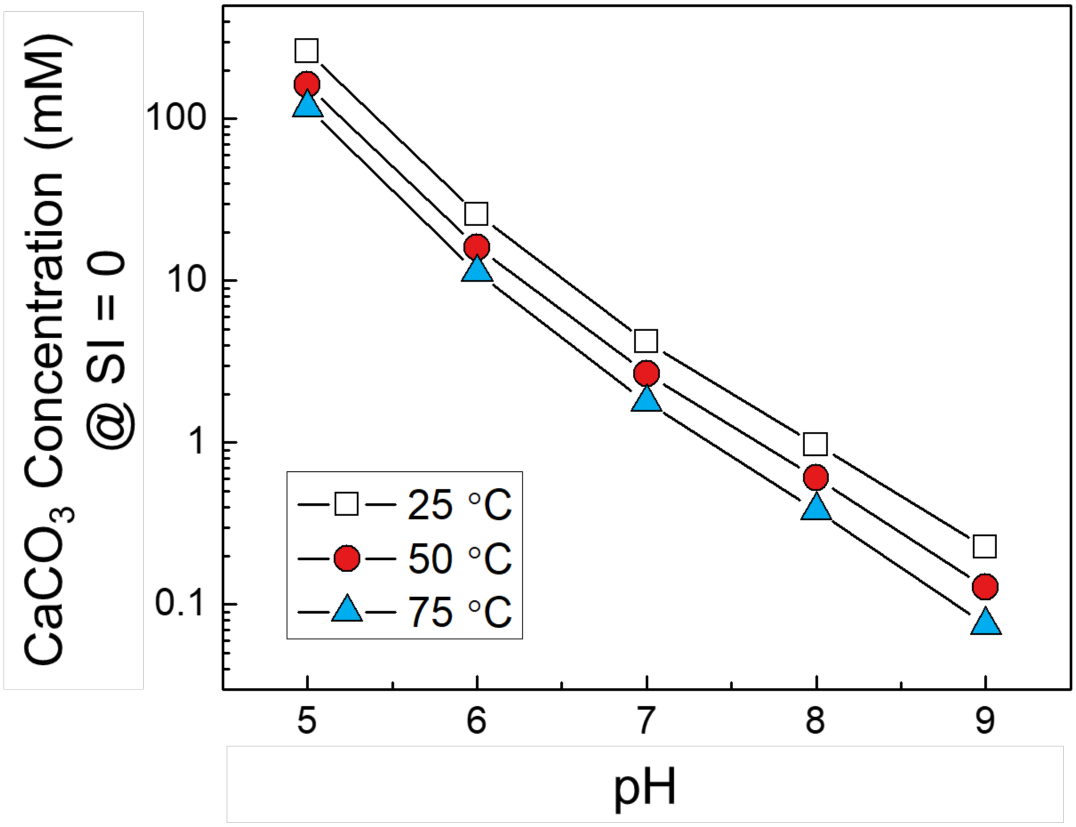

Figure S7 – Effect of temperature and pH on the equilibrium saturation index (SI) of calcium carbonate ($CaCO_3$)

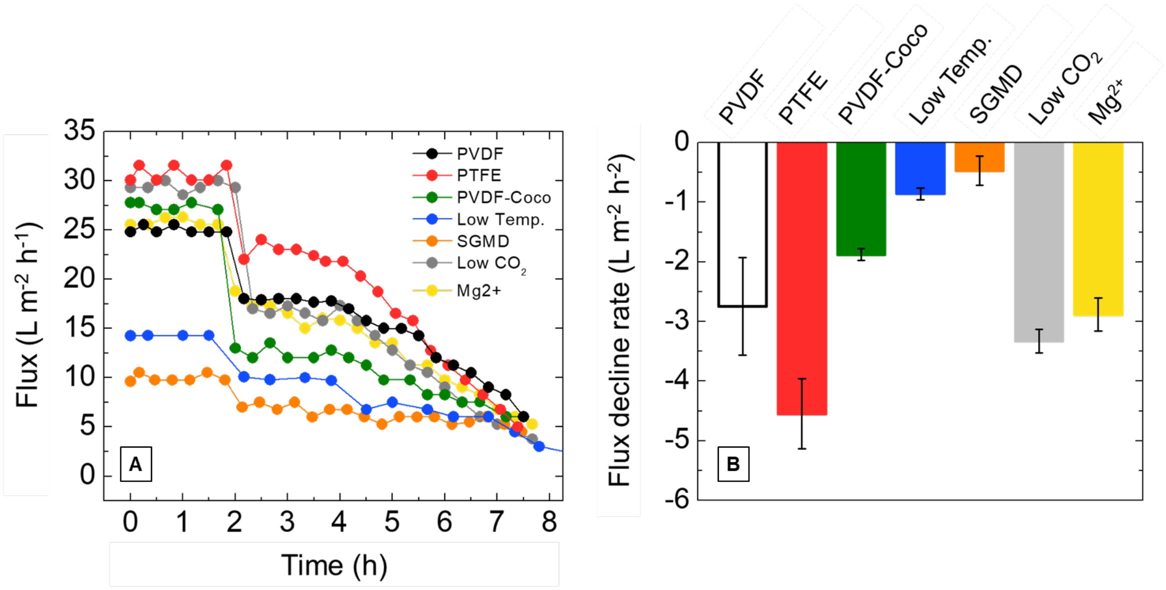

Figure S8 – (A) Flux vs. time and (B) flux decline rate for each of the experimental conditions tested.

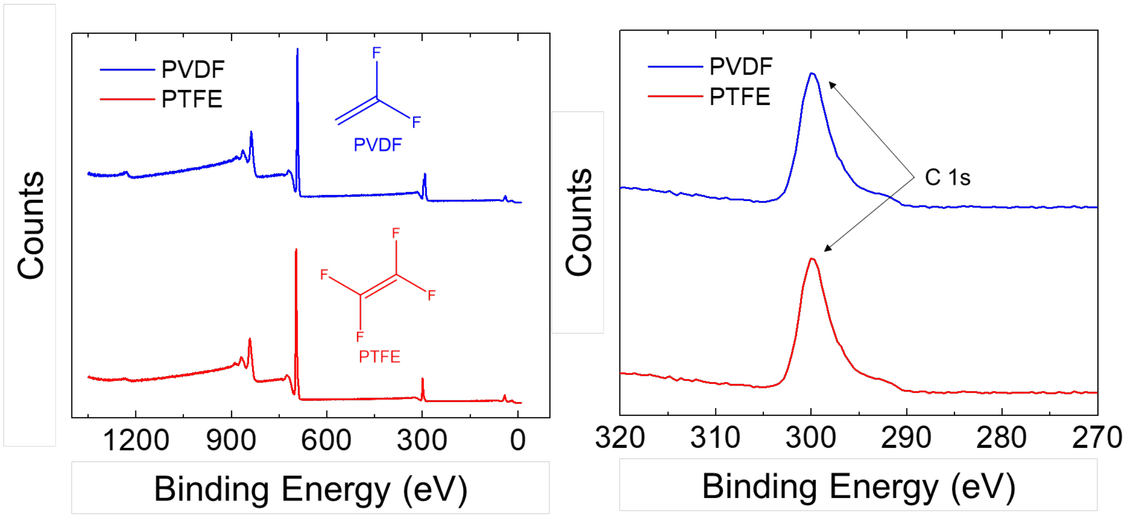

Figure S9 – X-ray photoelectron spectroscopy (XPS) showing the matching C 1s peaks of the pristine PVDF and PTFE membranes.

References:

(1) Zevenhoven, R.; Fagerlund, J. Mineralisation of Carbon Dioxide (CO2). In *Developments and Innovation in Carbon Dioxide (CO2) Capture and Storage Technology*; Elsevier, 2010; pp 433–462. https://doi.org/10.1533/9781845699581.4.433.

(2) Gadikota, G. Geo-Chemo-Physical Studies of Carbon Mineralization for Natural and Engineered Carbon Storage. Ph.D., Columbia University, United States -- New York. https://www.proquest.com/docview/1498550288/abstract/502DA4B14A794D17PQ/1 (accessed 2022-06-10).

(3) Ma, C.; Pietrucci, F.; Andreoni, W. Capture and Release of CO2 in Monoethanolamine Aqueous Solutions: New Insights from First-Principles Reaction Dynamics. *J. Chem. Theory Comput.* **2015**, *11* (7), 3189–3198. https://doi.org/10.1021/acs.jctc.5b00379.

(4) Mulder, M. *Basic Principles of Membrane Technology*; Springer Netherlands: Dordrecht, 1996. https://doi.org/10.1007/978-94-009-1766-8.

(5) Nguyen, D. T.; Lee, S.; Lopez, K. P.; Lee, J.; Straub, A. P. Pressure-Driven Distillation Using Air-Trapping Membranes for Fast and Selective Water Purification. *Sci. Adv.* **2023**, *9* (28), eadg6638. https://doi.org/10.1126/sciadv.adg6638.

(6) Razmjou, A.; Arifin, E.; Dong, G.; Mansouri, J.; Chen, V. Superhydrophobic Modification of TiO2 Nanocomposite PVDF Membranes for Applications in Membrane Distillation. *J. Membr. Sci.* **2012**, *415–416*, 850–863. https://doi.org/10.1016/j.memsci.2012.06.004.

(7) Boo, C.; Lee, J.; Elimelech, M. Omniphobic Polyvinylidene Fluoride (PVDF) Membrane for Desalination of Shale Gas Produced Water by Membrane Distillation. *Environ. Sci. Technol.* **2016**, *50* (22), 12275–12282. https://doi.org/10.1021/acs.est.6b03882.

(8) Jeon, C. W.; Park, S.; Bang, J.-H.; Chae, S.; Song, K.; Lee, S.-W. Nonpolar Surface Modification Using Fatty Acids and Its Effect on Calcite from Mineral Carbonation of Desulfurized Gypsum. *Coatings* **2018**, *8* (1), 43. https://doi.org/10.3390/coatings8010043.

(9) Razavi, S. M. R.; Oh, J.; Sett, S.; Feng, L.; Yan, X.; Hoque, M. J.; Liu, A.; Haasch, R. T.; Masoomi, M.; Bagheri, R.; Miljkovic, N. Superhydrophobic Surfaces Made from Naturally Derived Hydrophobic Materials. *ACS Sustain. Chem. Eng.* **2017**, *5* (12), 11362–11370. https://doi.org/10.1021/acssuschemeng.7b02424.

(10) Camacho, L. M.; Dumée, L.; Zhang, J.; Li, J.; Duke, M.; Gomez, J.; Gray, S. Advances in Membrane Distillation for Water Desalination and Purification Applications. *Water* **2013**, *5* (1), 94–196. https://doi.org/10.3390/w5010094.

(11) García-Payo, M. C.; Izquierdo-Gil, M. A.; Fernández-Pineda, C. Wetting Study of Hydrophobic Membranes via Liquid Entry Pressure Measurements with Aqueous Alcohol Solutions. *J. Colloid Interface Sci.* **2000**, *230* (2), 420–431. https://doi.org/10.1006/jcis.2000.7106.

(12) Kim, B.-S.; Harriott, P. Critical Entry Pressure for Liquids in Hydrophobic Membranes. *J. Colloid Interface Sci.* **1987**, *115* (1), 1–8. https://doi.org/10.1016/0021-9797(87)90002-6.

(13) Eykens, L.; Hitsov, I.; De Sitter, K.; Dotremont, C.; Pinoy, L.; Nopens, I.; Van der Bruggen, B. Influence of Membrane Thickness and Process Conditions on Direct Contact Membrane Distillation at Different Salinities. *J. Membr. Sci.* **2016**, *498*, 353–364. https://doi.org/10.1016/j.memsci.2015.07.037.
(14) Su, C.; Horseman, T.; Cao, H.; Christie, K.; Li, Y.; Lin, S. Robust Superhydrophobic Membrane for Membrane Distillation with Excellent Scaling Resistance. *Environ. Sci. Technol.* **2019**, *53* (20), 11801–11809. https://doi.org/10.1021/acs.est.9b04362.
(15) Xiao, Z.; Zheng, R.; Liu, Y.; He, H.; Yuan, X.; Ji, Y.; Li, D.; Yin, H.; Zhang, Y.; Li, X.-M.; He, T. Slippery for Scaling Resistance in Membrane Distillation: A Novel Porous Micropillared Superhydrophobic Surface. *Water Res.* **2019**, *155*, 152–161. https://doi.org/10.1016/j.watres.2019.01.036.
(16) Sparenberg, M.-C.; Chergaoui, S.; Sang Sefidi, V.; Luis, P. Crystallization Control via Membrane Distillation-Crystallization: A Review. *Desalination* **2021**, *519*, 115315. https://doi.org/10.1016/j.desal.2021.115315.
(17) Edwie, F.; Chung, T.-S. Development of Simultaneous Membrane Distillation–Crystallization (SMDC) Technology for Treatment of Saturated Brine. *Chem. Eng. Sci.* **2013**, *98*, 160–172. https://doi.org/10.1016/j.ces.2013.05.008.
(18) Tun, C. M.; Fane, A. G.; Matheickal, J. T.; Sheikholeslami, R. Membrane Distillation Crystallization of Concentrated Salts—Flux and Crystal Formation. *J. Membr. Sci.* **2005**, *257* (1), 144–155. https://doi.org/10.1016/j.memsci.2004.09.051.
(19) Jiang, X.; Shao, Y.; Sheng, L.; Li, P.; He, G. Membrane Crystallization for Process Intensification and Control: A Review. *Engineering* **2021**, *7* (1), 50–62. https://doi.org/10.1016/j.eng.2020.06.024.
(20) Lim, X. Could the World Go PFAS-Free? Proposal to Ban 'Forever Chemicals' Fuels Debate. *Nature* **2023**, *620* (7972), 24–27. https://doi.org/10.1038/d41586-023-02444-5.
(21) Bao, Y.; Deng, S.; Jiang, X.; Qu, Y.; He, Y.; Liu, L.; Chai, Q.; Mumtaz, M.; Huang, J.; Cagnetta, G.; Yu, G. Degradation of PFOA Substitute: GenX (HFPO–DA Ammonium Salt): Oxidation with UV/Persulfate or Reduction with UV/Sulfite? *Environ. Sci. Technol.* **2018**, *52* (20), 11728–11734. https://doi.org/10.1021/acs.est.8b02172.
(22) Langenbach, B.; Wilson, M. Per- and Polyfluoroalkyl Substances (PFAS): Significance and Considerations within the Regulatory Framework of the USA. *Int. J. Environ. Res. Public. Health* **2021**, *18* (21), 11142. https://doi.org/10.3390/ijerph182111142.
(23) Lohmann, R.; Cousins, I. T.; DeWitt, J. C.; Glüge, J.; Goldenman, G.; Herzke, D.; Lindstrom, A. B.; Miller, M. F.; Ng, C. A.; Patton, S.; Scheringer, M.; Trier, X.; Wang, Z. Are Fluoropolymers Really of Low Concern for Human and Environmental Health and Separate from Other PFAS? *Environ. Sci. Technol.* **2020**, *54* (20), 12820–12828. https://doi.org/10.1021/acs.est.0c03244.
(24) Krevelen, D. W. van; Nijenhuis, K. te. *Properties of Polymers: Their Correlation with Chemical Structure: Their Numerical Estimation and Prediction from Additive Group Contributions*, 4th, completely rev. ed ed.; Elsevier: Amsterdam, 2009.
(25) 1H,1H,2H,2H-Perfluorodecyltriethoxysilane. http://www.chemspider.com/Chemical-Structure.128237.html.
(26) Fisher, L. r.; Mitchell, E. e.; Parker, N. s. Interfacial Tensions of Commercial Vegetable Oils with Water. *J. Food Sci.* **1985**, *50* (4), 1201–1202. https://doi.org/10.1111/j.1365-2621.1985.tb13052.x.
(27) Coto, B.; Martos, C.; Peña, J. L.; Rodríguez, R.; Pastor, G. Effects in the Solubility of CaCO3: Experimental Study and Model Description. *Fluid Phase Equilibria* **2012**, *324*, 1–7. https://doi.org/10.1016/j.fluid.2012.03.020.
